# Effects of nitrogen addition and mowing on rodent damage in an Inner Mongolian steppe

**DOI:** 10.1002/ece3.3949

**Published:** 2018-03-23

**Authors:** Yinzhan Liu, Gaigai Ma, Zhiman Zan, Anqun Chen, Yuan Miao, Dong Wang, Renhui Miao

**Affiliations:** ^1^ International Joint Research Laboratory for Global Change Ecology State Key Laboratory of Cotton Biology School of Life Sciences Henan University Kaifeng Henan China; ^2^ Agricultural Schools Henan University of Science and Technology Luoyang China

**Keywords:** aboveground net primary productivity, burrow density, *Citellus dauricus*, community cover, semiarid grassland

## Abstract

Rodent damage is a serious threat to sustainable management of grassland. The effects of nitrogen (N) deposition and grassland management on rodent damage have been scarcely studied. Here, we reported the effects of 2 years of N addition and mowing on burrow density and damage area of *Citellus dauricus* in a semiarid steppe in Inner Mongolia. N addition significantly aggravated, while mowing alleviated rodent damage in the grassland under study. Burrow density and damage area increased 2.8‐fold and 4.7‐fold, in N addition plots compared to the ambient N addition treatment, respectively. Conversely, mowing decreased burrow density and damage area by 75.9% and 14.5%, respectively, compared to no mowing plots. Observed changes in rodent damage were mainly due to variations in plant community cover, height, and aboveground net primary productivity. Our findings demonstrate that N addition and mowing can affect the rodent density and activity in grassland, suggesting that the effects of a changing atmospheric composition and land use on rodent damage must be considered in order to achieve better grassland management.

## 1 INTRODUCTION

Grassland rodents, including voles, zokors, pikas, marmots, rabbits, and ground squirrels, play an important role in affecting ecosystem structure and function (Davidson et al., 2010; Jiang, Wang, Li, Shi, & Yang, 2017; Olofsson, Tømmervik, & Callaghan, 2012; Zhang, Zhong, & Fan, 2003). At moderate densities, rodents aid in maintaining diversity in the grassland, because their extensive excavations promote soil nutrient cycling and seed dispersal (Davidson et al., 2010); besides, they are a food resource to other animals in higher trophic levels (Delibesmateos, Smith, Slobodchikoff, & Swenson, 2011; Zhang, Zhang, & Liu, 2003). Additionally, their abandoned burrows provide shelter to native birds and lizards (Delibesmateos et al., 2011; Zhang, Zhang, & Liu, 2003). However, if their density exceeds the capacity of the ecosystem, rodents become a pest (Zhang, Zhong, & Fan, 2003). During outbreaks, rodents can destroy the grassland by foraging on aboveground parts, by cutting plant roots and burying plants under the soil they dig out (Li, Liu, Frelich, & Sun, 2011; Zhang, Zhong, & Fan, 2003).

Rodent damage to grasslands poses a serious threat to the sustainable development of pasture areas in China. Therefore, previous studies in the grasslands of China have focused on the methods to control rodent population and damage (Su et al., 2013; Zhang, Zhong, & Fan, 2003). Since the end of 20th century, the rodent‐infested area in the grassland of China showed a continuous annual growth of 10%–20% (Zhang, Zhong, & Fan, 2003), for an estimated at 40‐50 × 10^6^ tons of annual biomass loss caused by rodents (Zhang, Zhong, & Fan, 2003). According to the National Grassland Monitoring Report of China in 2016, the rodent‐infested area occupies 28.07 × 10^6^ ha, covering 7.1% of the total pasture area (Liu, 2017).

Previous studies have documented that rodent damage can be affected by factors influencing food sources and rodent predators. Indeed, most previous studies of rodent damage in grasslands have focused on the effects of grazing, climate warming, and changes in annual precipitation regime. For instance, rodent survival rates are reportedly higher under heavy grazed management (Harris, 2010; Li et al., 2016; Zhang, Pech, et al., 2003; Zhou, Zhao, Tang, Gu, & Zhou, 2005). Climate warming has aggravated rodent damage through promoting clonal growth of *Potentilla anserina* in an alpine meadow (Li et al., 2011). Abnormal annual precipitation could inhibit rodent outbreaks in the grassland (Zhang, Pech, et al., 2003). However, responses of rodent damage to changes in other environmental factors or land management practices, such as N deposition and mowing, have been rarely studied.

Increasing atmospheric N deposition is one of the most disturbance factors related to global change. The N deposition rate has increased 2–3 times since the industrial revolution, and is predicted to continuously increase during this century (Liu, Zhang, et al., 2013; Penuelas et al., 2013). Given that N is one of the main factors limiting plant growth (Lebauer & Treseder, 2008; Liu et al., 2017), N deposition can stimulate plant growth (Chang et al., 2016; Xia & Wan, 2008), which in turn affects animal communities (Cebrian, Kielland, & Finstad, 2008; Wimp, Murphy, Finke, Huberty, & Denno, 2010). Previous studies have reported that N addition can alter life history strategies (Parsons, Hellgren, Jorgensen, Leslie, & Benton, 2005) and food selection of the rodents (Yi, Li, Zhang, Zhang, & Wang, 2016), but the effect of nitrogen addition on rodent density or on the extent of damage they cause has not been documented.

Mowing has become a typical grassland management strategy in pasture areas (Cao, Li, & Yu, 2009; Han, Zhang, Wang, Jiang, & Xia, 2011; Jia, Shao, & Wei, 2012). As an important human activity, mowing can affect microclimate (Wan, Luo, & Wallace, 2002); it can alter plant functional traits (Liu et al., 2017) and increase species richness (Yang et al., 2012), with subsequently affecting the quality and quantity of food resources. Additionally, mowing can expose rodents to their predators, thereby reducing rodent damage (White, Horskins, & Wilson, 1998). Mowing reduced the population density of *Microtus*, but it increased that of *Peromyscus maniculatus*, in a tallgrass prairie in North America (Lemen & Clausen, 1984). It also increased the population size of *Sigmodon hispidus* and *Microtus ochrogaster*, but did not affect populations of *Peromyscus leucopus* and *P. maniculatus*, in an old grassland in eastern Kansas (Slade & Crain, 2006). Thus, the impacts of mowing on rodent damage remain controversial. Furthermore, N addition and mowing have interactive effects on plant growth (Liu et al., 2017), which may affect rodent damage. However, to the best of our knowledge, interactive effects of N addition and mowing on rodent damage have not been reported.

Therefore, we conducted an in situ experiment involving N addition and mowing in a semiarid steppe in Duolun County in the Xilingol grassland, and investigated burrow density and percentage of rodent damage area, and plant community parameters including cover, height, aboveground net primary productivity, Shannon–Wiener diversity index, Simpson dominance index, and evenness in the experiment. We hypothesized that N addition would aggravate, whereas mowing would alleviate rodent damage, since N addition increases, while mowing decreases aboveground biomass, which is the food source used by rodents. Furthermore, with the interactive effect between N addition and mowing on plant growth in mind (Liu et al., 2017), we hypothesized that N and mowing would interactively affect rodent damage.

## 2 MATERIALS AND METHODS

### 2.1 Description of the study site

This study was performed in a temperate steppe in an ecotone of agriculture–animal husbandry in Duolun County (42°02′N, 116°17′E, 1,324 m a.s.l.), in the Xilingol grassland, Inner Mongolia, China. The Xilingol grassland is an important pasture area and ecotone between agriculture and animal husbandry in China. It covers 2.0 × 10^5^ km^2^ (Han, Owens, Wu, & Huang, 2009) and supports a human population of 2.1 × 10^5^ (Shiyomi et al., 2011). It is estimated that 64% of the Xilingol grassland is degraded; thus, forage quality has drastically decreased in the region (Han et al., 2009). Rodent damage is one of the most important factors underlying degradation in the Xilingol grassland. The rodent damage area amounts to 2–3 × 10^4^ km^2^, causing severe economic losses (Zhang, Yang, Wang, Cai, & Qiao, 2014). The N deposition rate has reached 9.24 kg N ha^−1^ year^−1^ and steadily increases at a rate of 0.42 kg N ha^−1^ year^−1^ in this area (Liu, Zhang, et al., 2013; Lü & Tian, 2007). Mowing is the only management approach for the local herdsmen to store forage in the nongrowing season (Cao et al., 2009). However, the effects of N addition and/or mowing on rodent damage in this area have not yet been investigated. The mean annual temperature in the steppe is approximately 2.1°C, with temperature ranging from −17.5°C in January to 18.9°C in July. Mean annual precipitation is approximately 383 mm, 90% of which falls in the growing reason from May to October. The soil is chestnut‐type (Calcic Kastanozems). The plant community is dominated by perennial bunchgrasses, including *Stipa krylovii* and *Agropyron cristattum*, and subshrubs such as *Artemisia frigida*. Perennial rhizome grasses, including *Aneurotepidimu chinense* and *Carex duriuscula* subsp. *rigescens*, and perennial forbs, including *Heteropappus altaicus*,* Melissitus ruthenica*, and *Potentilla tanacetifolia*, are also abundant in the steppe. The steppe was fenced in 2001 to exclude large herbivores including horses, cattle, sheep, and other livestocks, while the feeding activity of small herbivores, including rabbits, voles, and ground squirrels, continues unchecked.

**Figure 1:**
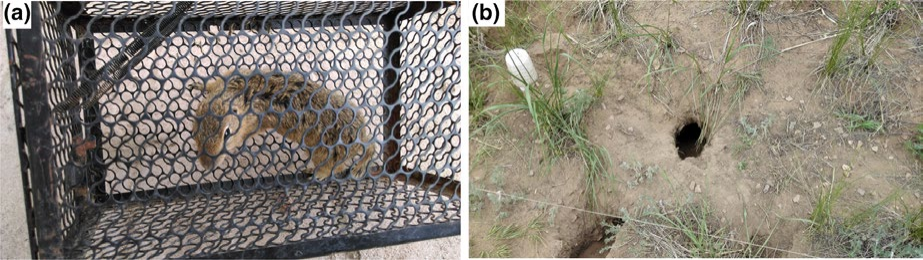
Photographs of rodent and mousetrap (a); entrance to a burrow (b) in the experimental site

The dominant rodent species in the steppe are Brandt's vole (*Lasiopodomys brandtii*), the Chinese striped hamster (*Cricetulus barabensis*), and the ground squirrel (*Citellus dauricus*). To identify the species composition in the experimental site, rodents in a 150 m × 150 m area around the study site, although not included in it, were trapped with mousetraps at the beginning of the experiment (Figure 1a).They were all identified according to the Chinese Zoological Illustration (Zhang & Ye, 1988) and Pest Management (Guo et al., 2005), and then released. Only ground squirrel (*C. dauricus*) was trapped during the experimental period. *C. dauricus* are the common rodents in the steppe. They are morphologically and behaviorally similar to rats. Their diets are the green parts of most plant species in the steppe, including *A. frigida*,* S. krylovii*, and *M. ruthenica* (Liu, Wang, Wang, Han, et al., 2013). Ground squirrels live underneath the ground, in burrow systems, and push loosened soil to the surface, thus destroying the grassland by burying the plants (Liu, Wang, Wang, Han, et al., 2013). Their population density was estimated to have reached 6.5 heads per ha in the Inner Mongolian typical grassland (Liu, Wang, Wang, Zhang, et al., 2013).

### 2.2 Experimental design

The experiment was laid out in a factorial design with two factors, each with two levels: N addition (ambient N deposition and ambient plus 10 g N m^−2^ year^−1^) and mowing (no mowing, mowing). Thus, there were four treatments, including (1) control (C, ambient N deposition and no mowing), (2) mowing (M), (3) N addition (N, ambient plus 10 g N m^−2^ year^−1^), and (4) combined mowing with N addition (MN). In the N addition plots, N was added as NH_4_NO_3_ at a rate of 10 g N m^−2^ year^−1^, once a year in early June, since 2013. Mowing treatment clipped and removed aboveground biomass at the height of 5 cm, once a year in early September, since 2012. Each treatment was replicated five times, with each plot being 4 × 4 m^2^.

### 2.3 Soil temperature and moisture

**Figure 2:**
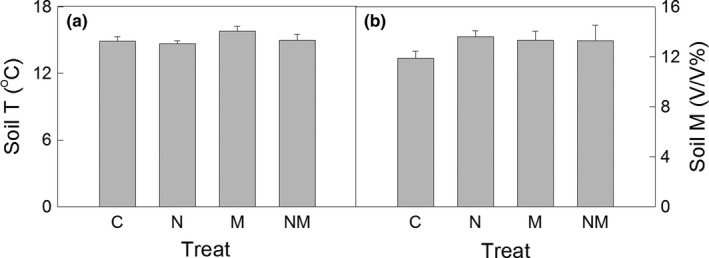
Soil temperature (a, Soil T) and moisture (b, Soil M) under the different treatments. C: control, N: N addition, M: mowing, NM: N addition + mowing

Soil temperature at the depth of 10 cm was measured three times a month, with a thermocouple probe (Li‐8100‐201, Li‐Cor Inc., Lincoln, NE, USA) in 2014. Soil temperature in each plot was measured at three random points in each measurement. Soil moisture at a depth of 10 cm was measured four times a month, with portable time domain reflectometer equipment (TDR200; Soil Moisture Equipment Corp., Santa Barbara, CA, USA). As in soil temperature measurement, soil moisture was probed randomly at three points in each plot. Neither soil temperature nor moisture was affected by N addition, mowing, or their interaction (Table 1, Figure 2).

**Table 1:** Effects of mowing (M), N addition (N), and their interaction on soil temperature, soil moisture, plant community cover, density, species richness, community height, ANPP, Shannon–Wiener index, dominance, evenness, burrow density, and damage area, as analyzed by two‐way ANOVA ^, p < 0.1; *, p < 0.05; **, p < 0.001

	M	N	M * N
Soil temperature	1.831	1.486	0.427
Soil moisture	0.473	1.065	1.257
Community cover	2.737	5.407*	1.682
Density	4.915*	0.108	2.876
Species richness	0.031	0.031	0.763
Community height	4.102^	7.053*	1.243
ANPP	4.708*	2.482	0.166
Shannon–Wiener index	0.076	1.906	5.958*
Dominance	0.043	0.043	0.681
Evenness	0.014	2.283	2.283
Burrow density	40.590***	36.860***	6.882*
Damage area	19.537***	21.760***	6.215*

### 2.4 Ground squirrel burrow density and damage area percentage

Burrow density in the steppe remained at about 20 ha^−1^ from 2010 to 2013; however, it increased to 375 ± 190 ha^−1^ in 2014, ranging from 60 ha^−1^ to 1,500 ha^−1^ (observed by Liu and Ma). Nonetheless, no burrow was found before 2014 within the experimental site (Observed by Liu and Ma). Burrow density and damage area percentage were evaluated in 2014 following the methods used in a previous study in a Tibetan alpine meadow (Li et al., 2011). The number of the burrow entrances (Figure 1b) was counted in late July, when burrow number peaks. The loosened soil‐buried area in each plot was divided into many rectangles and triangles. The total area of all the rectangles and triangles was added together and then divided by the total plot area (16 m^2^) to calculate damage area percentage.

### 2.5 Plant community survey

All community parameters were monitored at peak plant biomass in early August 2014. One 1 × 1 m^2^ quadrat was established at each plot in 2005, and a 1 × 1 m^2^ steel frame with 10 × 10 cm^2^ grid squares was installed above the canopy of each quadrat to facilitate cover estimation. Species cover for each species in all the grids was visually estimated, and community cover was calculated by the sum of all species covers. The number of individuals of each species in the quadrat was counted as the density at the species level, and the densities of all species in the quadrat were added together as the community plant density. Species richness was recorded as the number of plant species in the quadrats. Plant height of each species was randomly measured on three stems in the quadrat and averaged. The community height was calculated following a previous report (Liu, Mu, Niklas, Li, & Sun, 2012).

After estimations of cover, density, species richness, and height were completed, aboveground living biomass of each species within the quadrat was clipped and oven‐dried to constant weight at 65°C. Total dry biomass for each plot was calculated as the aboveground net primary productivity (ANPP). Shannon–Wiener diversity index, Simpson dominance index and evenness of every plot were calculated based on biomass data of plant community in each quadrat.

### 2.6 Data analysis

Effects of mowing and N addition on soil microclimate (temperature, moisture), plant community (community cover, plant density, species richness, community height, ANPP, Shannon–Wiener diversity index, Simpson dominance index, and evenness), and rodent damage (burrow density and damage area percentage) were analyzed with two‐way ANOVAs. Post hoc LSD tests were employed to examine differences among the four treatments. Linear regression was used to test the relationships between rodent damage parameters and plant community and soil microclimate. All analyses were conducted with SPSS package 16.0 (SPSS Inc., Chicago, IL, USA).

## 3 RESULTS

### 3.1 Ground squirrel damage

**Figure 3:**
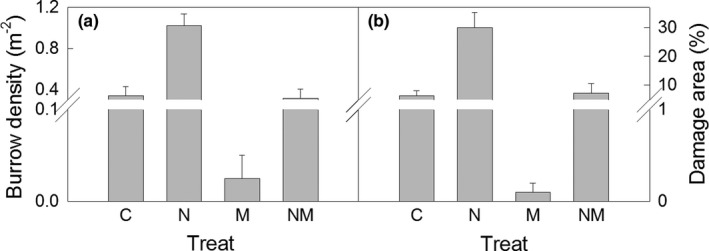
Burrow density (a) and damage area percentage (b) under the different treatments. C, control; N, N addition; M: mowing, NM: N addition + mowing

Nitrogen addition significantly enhanced burrow density by 2.8‐fold (*p *<* *.05, Figure 3a), while mowing significantly decreased it by 75.9% (relative difference, Table 1, Figure 3a). Additionally, we observed significant interactive effects of N addition and mowing on burrow density and damage area percentage. N addition increased burrow density by 0.73 m^−2^ in the nonmown plots (post hoc, *p *<* *.05, Figure 3a) but enhanced it by only 0.29 m^−2^ in the mown plots (post hoc, *p *<* *.05, Figure 3a). Mowing decreased burrow density by 0.31 m^−2^ in the plots without N addition (post hoc, *p *<* *.05, Figure 3a), but decreased it by 0.75 m^−2^ in the nitrogen addition plots (post hoc, *p *<* *.05, Figure 3a).

Damage area increased 4.7 times by N addition (*p *<* *.05, Figure 3b), whereas it decreased by only 14.5% due to mowing (absolute difference, Table 1, Figure 3b). Furthermore, significant interactions were found between N addition and mowing on damage area percentage (Table 1). N addition enhanced the damage area percentage by 23.4% in the nonmown plots (absolute difference, post hoc, *p *<* *.05, Figure 3b), but only increased it by 7.1% in the mown plots (post hoc, *p *<* *.05, Figure 3b). On the other hand, mowing reduced the damage area percentage by 6.3% in the ambient N deposition plots (absolute difference, post hoc, *p *<* *.05, Figure 3b) and decreased it by 22.6% in the N addition plots (absolute difference, post hoc, *p *<* *.05, Figure 3b).

### 3.2 Plant community

**Figure 4:**
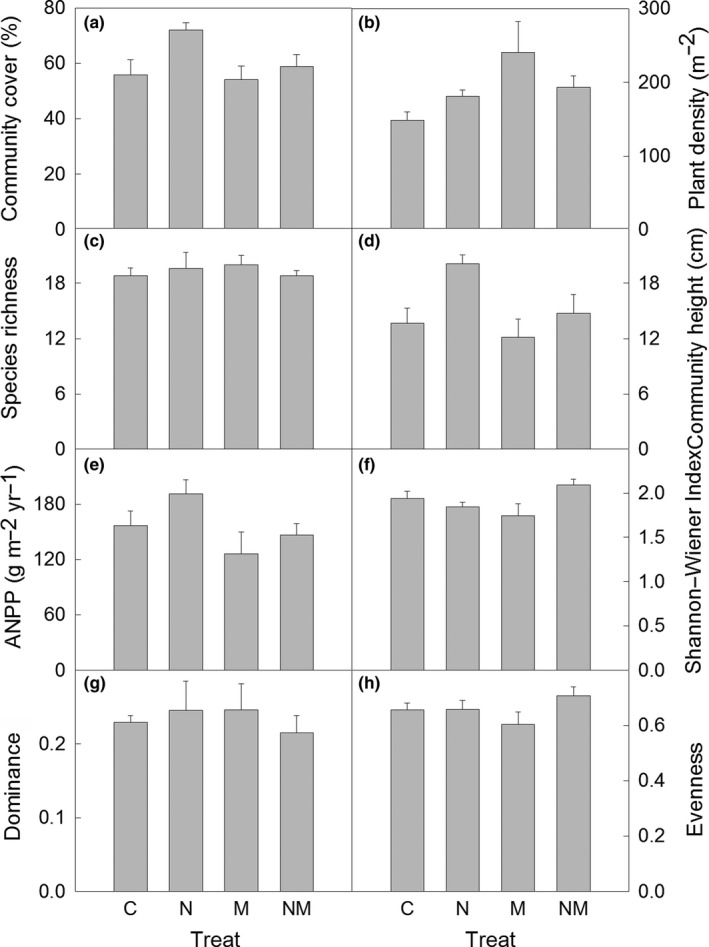
Plant community cover (a), density (b), species richness (c), community height (d), ANPP (e), Shannon–Wiener index (f), dominance (g), and evenness (h) under the different treatments. C, control; N, N addition, M, mowing; NM: nitrogen addition + mowing

Nitrogen addition significantly elevated community cover by 10.4% (absolute difference, Table 1, Figure 4a). In contrast, mowing did not alter community cover (Table 1, Figure 4a), although it significantly reduced plant density by 31.5% (Table 1, Figure 4b). On the other hand, species richness was not altered by N addition, mowing, or their combination (Table 1, Figure 4c). Community height was significantly elevated (4.6 cm, *p *<* *.05) by N addition, but marginally reduced (3.6 cm, .05 < *p *<* *.1) by mowing (Table 1, Figure 4d). Community height was significantly higher under the nitrogen addition treatment (20.1 cm) than that under the control (13.7 cm), mowing (12.1 cm), or combination (14.7 cm) treatments (post hoc, *p *<* *.05, Figure 4d). Mowing significantly reduced ANPP by 21.7% (Table 1, Figure 4e). No other significant effects of N addition, mowing, or their interaction were found on any of the aforementioned community parameters (Table 1).

The Shannon–Wiener diversity index, the Simpson dominance index, and evenness were not affected by N addition or mowing (Table 1, Figure 4f–h). However, a significant interactive effect by nitrogen addition and mowing on the Shannon–Wiener diversity index was detected (Table 1). N addition significantly reduced the diversity index by 0.35 in the mown plots, but not in the nonmown plots (Figure 4f).

### 3.3 Relations between ground squirrel damage, soil microclimate, and plant community parameters

**Figure 5:**
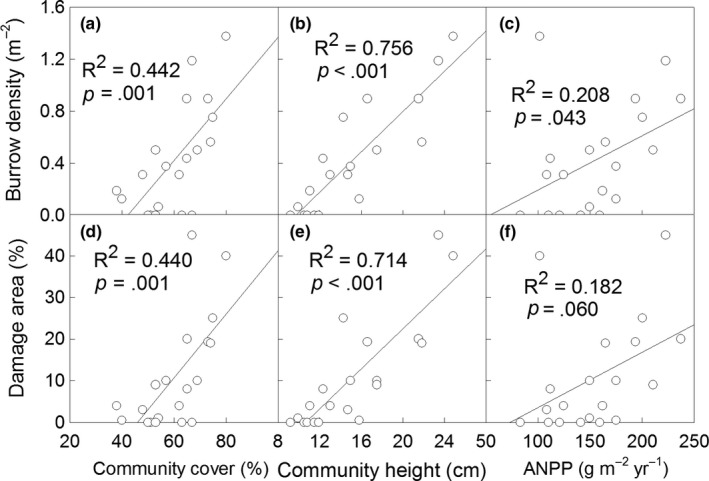
Correlation between burrow density and community cover (a), community height (b), and ANPP (c); correlation between damage area percentage and community cover (d), community height (e), and ANPP (f). Data points in each scatter diagram represent the values in experimental plot. Data of burrow density was lg‐transformed in the figure

Burrow density was positively and linearly correlated with community cover (*R* ^2^ = .442, *p *=* *.001, Figure 5a), community height (*R* ^2^ = .756, *p *<* *.001, Figure 5b), and ANPP (*R* ^2^ = .208, *p *=* *.043, Figure 5c). Damage area percentage significantly increased with increasing community cover (*R* ^2^ = .440, *p *=* *.001, Figure 5d) and community height (*R* ^2^ = .714, *p *<* *.001, Figure 5e); additionally, it marginally increased with increasing ANPP (*R* ^2^ = .182, *p *=* *.060, Figure 5f).

## 4 DISCUSSION

Burrow density reached 0.34 m^−2^ (i.e., 3,400 ha^−1^, Figure 3a) in the study site in 2014, a substantially higher value than that reported in previous studies (Liu, Wang, Wang, Han, et al., 2013; Liu, Wang, Wang, Zhang, et al., 2013; Zhang, Zhong, & Fan, 2003). Thus, we may conclude that the steppe suffered serious rodent damage. There may be two reasons for the higher density of *C. dauricus* observed in the study. First, the steppe is dominated by *A. frigida*, which occupied 30%–50% of biomass in the community (Yang et al., 2011), and happens to be the main diet for *C. dauricus* (Guo et al., 2005; Liu, Wang, Wang, Zhang, et al., 2013). Second, the study site is adjacent to a cropping area cultivated with potatoes, which provides abundant food for the rodents. Additionally, previous studies have reported that *C. dauricus* is a common rodent species in the steppe, but is less destructive than other rodent species, such as Brandt's vole (*Lasiopodomys brandtii*), the Chinese striped hamster (*Cricetulus Barabensis*), and the Mongolian gerbils (*Meriones unguiculatus*) (Liu, Wang, Wang, Han, et al., 2013; Liu, Wang, Wang, Zhang, et al., 2013; Zhang, Zhong, & Fan, 2003). Thus, *C. dauricus* is not considered as the dominant rodent pest in the Inner Mongolian grassland (Harris, 2010; Zhang, Zhong, & Fan, 2003). Nevertheless, our results suggested that *C. dauricus* can cause drastic disturbance in the grassland, and therefore, the damage induced by this species should be considered in the ecotone of agriculture–animal husbandry.

Multiple previous studies suggested that the abundance of herbivores increased with N addition (Bowdish & Stiling, 1998; Wimp et al., 2010); however, those results mainly concerned the effects of N addition on the abundance of insects. Our study showed that N addition increased both, burrow density and damage area. These observations support our hypothesis and confirmed that outbreaks of small mammals may result from N addition, suggesting that N deposition‐induced rodent damage must be considered in the future. There may be at least three potential causes for the outbreak of *C. dauricus* in the present study. First, N addition stimulated plant growth, thereby increasing the food resources for herbivores (Nijssen, Wallisdevries, & Siepel, 2017; Ritchie, 2000). In this aspect, although N addition did not significantly increase ANPP in the present study, ANPP showed an increasing trend (Figure 4e). Second, a taller plant community and a higher provided increased opportunity for the rodents to hide and escape from predators, mainly night owls and hawks (Andrey, Humbert, Pernollet, & Arlettaz, 2014; Woodcock & Pywell, 2009). Third, as we did not record data on feeding behavior by *C. dauricus*, we cannot exclude effects of N addition on food quality and intake selectivity by the rodents, as has been reported in a previous study (Yi et al., 2016).

Our study showed that mowing reduced both burrow density and damage percentage of *C. dauricus*, which further supports our hypothesis. These results are consistent with previous studies by Lemen and Clausen (1984), and by Edge, Wolff, and Carey (1995); however, the phenomenon disagrees with results observed by Jacob (2003), and by Slade and Crain (2006). In contrast to the positive effects of N addition, mowing reduced the ANPP (Figure 4e), consequently reducing the food supply, which in turn resulted in the reduction in rodent population density. Moreover, mowing may also decrease the standing litter height and alter the microclimate, which in turn would alter the plant growth strategy and decrease plant height (Klimesova, Latzel, de Bello, & van Groenendael, 2008; Liu et al., 2017). Actually, plant community height in the mown plots was marginally lower than that in the nonmown plots (Figure 4d), which we attributed to changes in growth strategy, considering the unchanged microclimate in our study. A shorter plant community height increases the risk of exposure to predators (Andrey et al., 2014; Ritchie, 2000; Woodcock & Pywell, 2009); therefore, rodents may choose taller plant communities to dig their burrows. The results indicate that mowing may be an effective method for controlling rodent damage in grasslands.

In addition, our observation that N addition‐induced outbreak of rodent damage was alleviated by mowing in the steppe supports our hypothesis about the interaction between N addition and mowing on rodent damage. The reason for this may be attributed the interactive effect between N addition and mowing on plant growth, which has been reported in a previous study (Liu et al., 2017). Actually, the plant community was much shorter in the nitrogen addition plots than in plots where no N was added (Figure 4d). Given that the effect of N addition on rodent damage was largely attributed to the increase in plant community height, mowing should partially suppress the effect of N addition on rodent damage, as, in fact, was recorded.

## 5 CONCLUSION

Our results showed that both N addition and mowing significantly altered rodent damage caused by the variation caused in plant community parameters. As rodents are the dominant pest in the northern grasslands of China, variation in rodent damage (e.g., burrow density and damage area) may result in changes in plant community structure and soil texture and structure, thereby affecting grassland productivity. Our study suggests that mowing can alleviate rodent damage and indicates that the impact of the changing atmospheric composition on rodent damage can be controlled by proper land‐use management in the semiarid grassland.

## CONFLICT OF INTEREST

None declared.

## AUTHORS CONTRIBUTION

Yinzhan Liu proposed the idea and designed the experiments; Gaigai Ma, Anqun Chen, and Yuan Miao performed the experiments; Zhiman Zan conducted the data analysis; Yinzhan Liu wrote the manuscript; Dong Wang and Renhui Miao edited the manuscript; and Yinzhan Liu and Gaigai Ma contributed equally in this work.
